# Supplementary material for: The systemic impact of deplatforming on social media
Source: PNAS Nexus. 2023 Oct 25;2(11):pgad346. doi: 10.1093/pnasnexus/pgad346 (PMC10638500; doi:10.1093/pnasnexus/pgad346)
Supplement: pgad346_Supplementary_Data [file pgad346_supplementary_data.pdf]

Supplementary Information: The Systemic Impact of Deplatforming on Social Media

Amin Mekacher[1,†], Max Falkenberg[1,†,*] and Andrea Baronchelli[1,2,*]

[1] *City University of London, Department of Mathematics, London EC1V 0HB, (UK)*
[2] *The Alan Turing Institute, British Library, London NW1 2DB, (UK)*
[†]These authors contributed equally.
[*]Corresponding authors: max.falkenberg@city.ac.uk, abaronchelli@turing.ac.uk
(Dated: October 4, 2023)

A. Manual account labelling

Our primary analysis heavily relies on Gettr users voluntarily disclosing their corresponding Twitter accounts, enabling us to conduct a cross-platform comparison of their activities. To ensure the accuracy of account matching, we manually examined the 300 accounts with the highest follower count on Gettr, out of the 1588 matched users we have in the cohort. The main aim is to confirm that the accounts on both platforms belong to the same person. Manual matching is a subjective judgement based on whether an account uses the same, or similar, handle on both platforms, the same, or similar, profile picture, and uses the same, or similar, profile biography. Out of the 300 accounts checked manually, 285 were successfully matched, corresponding to a 95% match rate. This match rate is comparable to the match rate found in previous studies using a similar method [1].

Following Elon Musk's amnesty on suspended accounts on Twitter announced in November 2022 [2], we also manually checked the 100 banned accounts with the highest follower count on Gettr. 33 of these accounts have since seen their ban being lifted on Twitter, thus confirming that Elon Musk did indeed offer such an amnesty to banned users. Interestingly, we noticed that many of these accounts share very similar narratives, primarily centered to Donald Trump's political propaganda and conspiracy theories revolving around Covid-19. Due to changes in the policy related to the Twitter API, we cannot study the activity of these accounts on Twitter after their ban has been lifted.

B. User acquisition and activity

One of the main findings of our analysis is that banned users are, on average, more active than non-verified or matched users, a first indicator that the banned cohort is more likely to benefit from being able to register on fringe platforms with a less stringent moderation policy. In particular, we notice that matched users are seven times more active on Twitter than on Gettr, wheras banned users are five times more active than matched users on Gettr. To ensure that our results are not conflated with the presence of various linguistic communities in the platform, we run the same analysis separately for the two major demographics on Gettr, i.e. by considering only the English- and Portuguese-speaking users. The results for the English-speaking community are displayed on figure 1, and show very similar trends than when considering the platform as a whole. Most notably, we still notice a peak of registrations in January 2022, related to Joe Rogan announcing his account on Gettr and which had a large impact on US-based users. Figure 1B also shows a larger propensity for banned users to be active on Gettr, when compared to matched and non-verified users.

Figure 2 shows the same results with the Portuguese-speaking cohort. An interesting observation on figure 2A is the absence of a peak of registrations in January 2022. This suggests that Joe Rogan's social media outreach mostly consists of English-speaking users, and therefore his registration on Gettr did not lead to a surge of Portuguese-speaking users on Gettr. However, there is a noticeable peak of registrations in September 2021, which is the result of Gettr's involvement in the Brazil CPAC 2021, with its CEO Jason Miller traveling to Brazil to attend the event [3]. Figure 2B highlights how banned users within the Brazilian community are also more active on Gettr than the other cohorts.

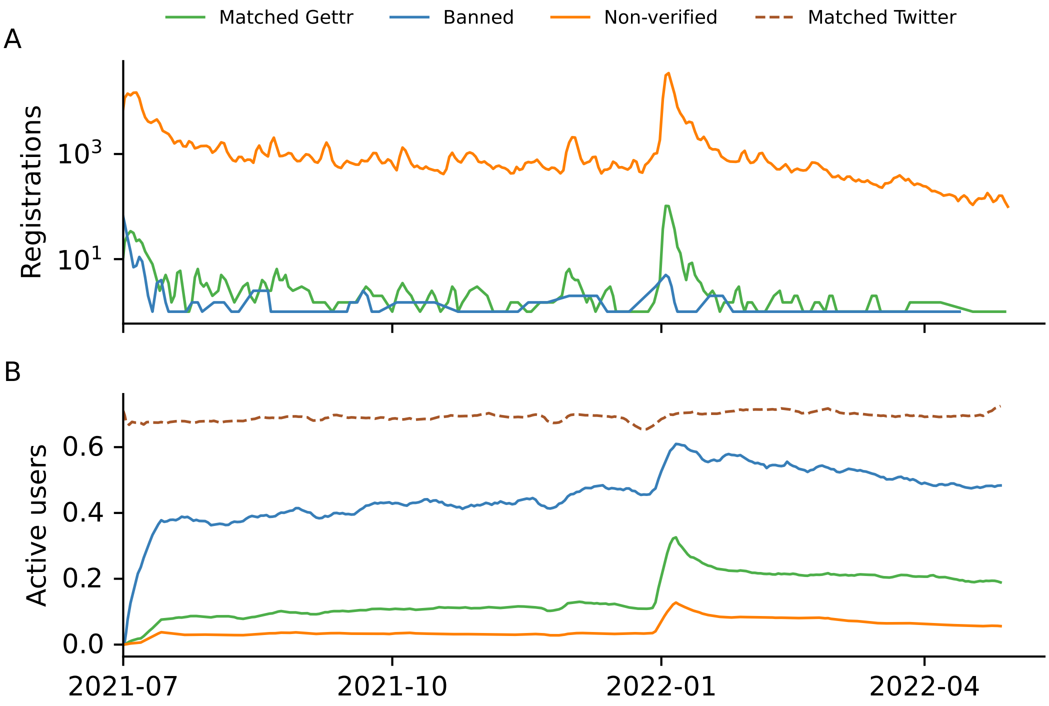

FIG. 1. **User registrations and daily activity for each cohort - English-speaking cohort.** (**a**) 3-day moving average of the daily number of users who registered on Gettr. The curve is displayed separately for the banned cohort (blue), the matched cohort (green) and other users who are not-verified on Gettr (orange). (**b**) 7-day moving average of the proportion of users from each cohort who were active on Gettr on a given day. The percentage of the matched cohort active on Twitter is also shown (dashed brown). Only English-speaking users are considered for this analysis.

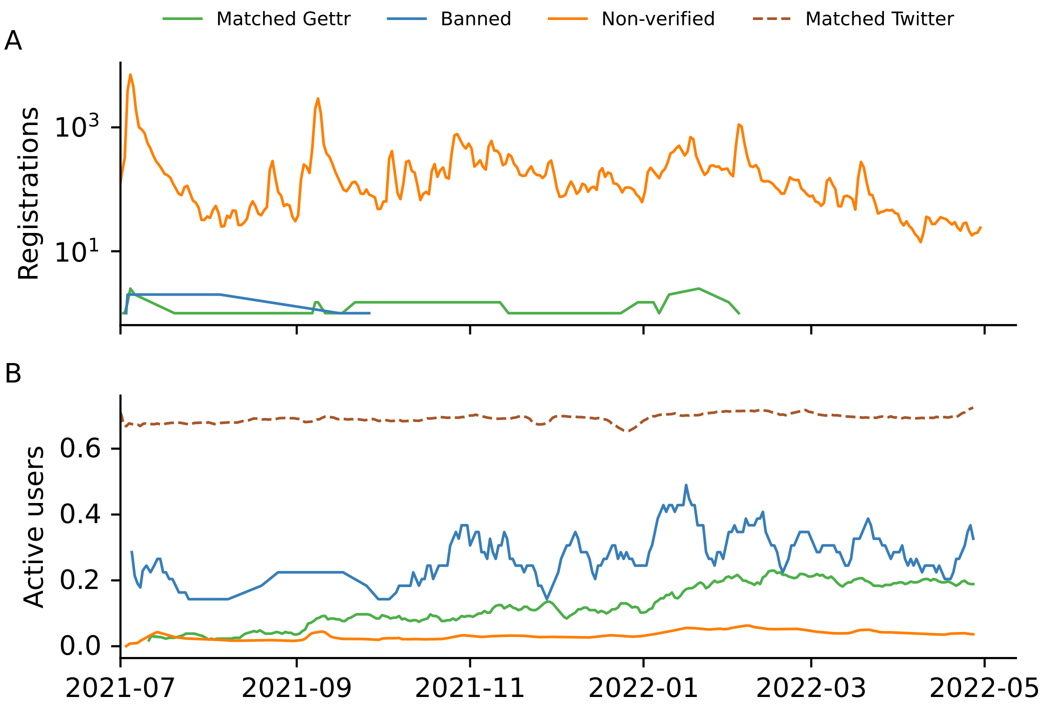

FIG. 2. **User registrations and daily activity for each cohort - Portuguese-speaking cohort.** (**a**) 3-day moving average of the daily number of users who registered on Gettr. The curve is displayed separately for the banned cohort (blue), the matched cohort (green) and other users who are not-verified on Gettr (orange). (**b**) 7-day moving average of the proportion of users from each cohort who were active on Gettr on a given day. The percentage of the matched cohort active on Twitter is also shown (dashed brown). Only Portuguese-speaking users are considered for this analysis.

C. User retention on Gettr

Our main analysis focuses on the two months with the highest numbers of new registrations: July 2021, when Gettr was first launched, and January 2022, when Joe Rogan joined the platform and led to almost a million new users to sign up as well. To ensure that our results also hold for any other month in our dataset, we compute the Kaplan-Meier estimate for every month up to April 2022, by separating the user cohorts mentioned in the main analysis (banned, matched and non-verified users). The results of the analysis are shown in figure 3. We notice that banned users are more likely to be active on the platform for a longer period of time, regardless of the considered registration month, whereas users in the matched and non-verified cohorts become inactive shortly after joining Gettr.

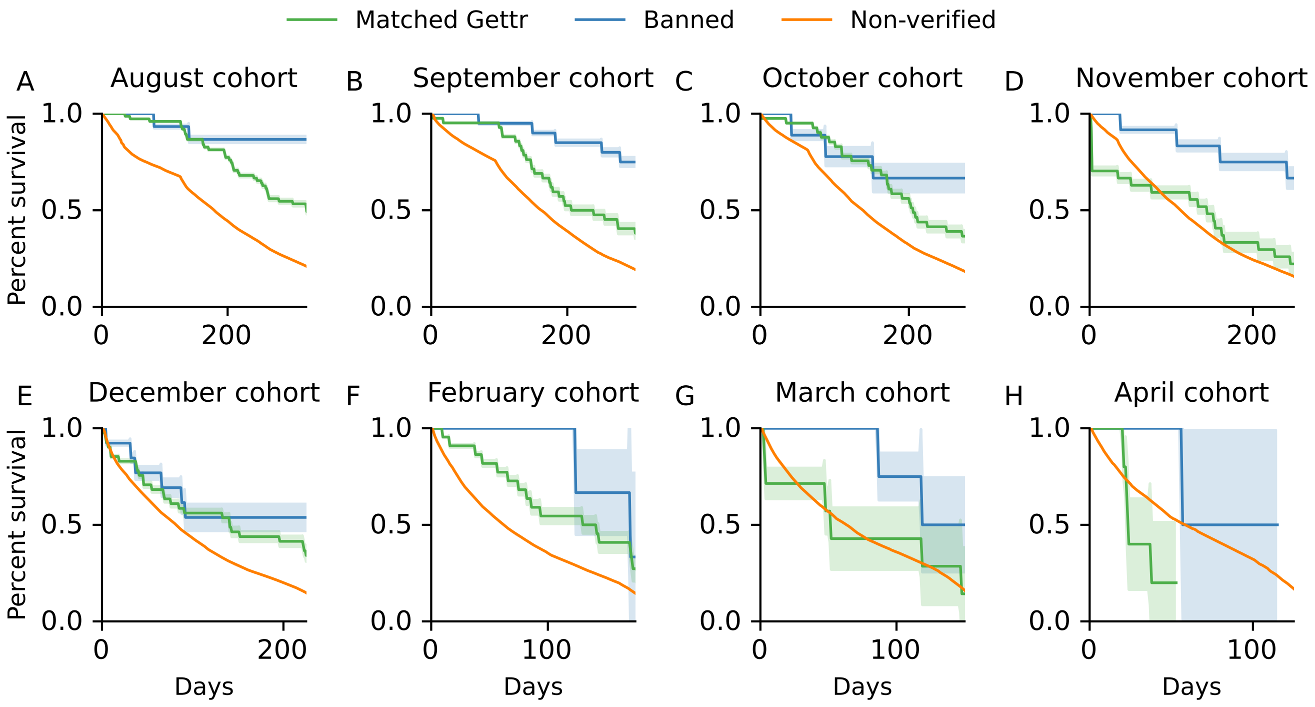

FIG. 3. **User retention for other registration months** (A) Kaplan-Meier survival curves for each user cohort showing the fraction of accounts who registered in August 2021 who remain active on Gettr a given number of days after registration for the banned cohort (blue), matched cohort (green) and the non-verified cohort (orange). The standard error of each curve is computed using Greenwood's formula [4] (see Methods). (B-H) Survival curves for other registration months, between September 2021 and April 2022 (excluding January 2022)

D. English-language Topic Modelling

To better understand Gettr content we employ a topic model, trained on English language Gettr posts using BERTopic [5] (see Methods), to characterise key discussion themes on the platform. The top 20 topics of classified Gettr content are shown in Table I alongside a representative Gettr post for each topic extracted automatically by BERTopic. Using the same model, we classify matched Twitter content using the same model; the promimence of each topic on both Gettr and Twitter is shown in Table II, with the ratio column indicating whether a topic is over- or under-represented on Gettr relative to Twitter. We list the median toxicity of posts classified as part of that topic on Gettr and on Twitter in table III.

The topic modelling reveals that content on Gettr is dominated by topics of significant relevance to US politics, and in particular of relevance to the political right. Topic 1 (Covid-19, vaccines) corresponds to approximately one sixth of all classified content on Gettr, with this value approaching a third in some months (see supplement). Topic 2 focuses explicitly on discussions around major social media platforms referencing the political right being deplatformed from Facebook and from Twitter.

It is worth considering those topics which are found disproportionately on Gettr, relative to the matched Twitter dataset. This includes topics related to the war in Ukraine (No. 3), the Canada Convoy protests which related to Covid-19 vaccine mandates (No. 4), and the 2020 US election and alegations of voter fraud, particularly in Arizona (No. 10). These are topics which are known to have been targets of the Twitter content moderation team; several accounts were suspended for (1) sharing pro-Russian content during the Ukraine war [6], (2) sharing Covid-19 misinformation [7], and (3) for supporting the January 2021 insurrection following the 2020 US elections [8]. Consequently, it is not surprising that these topics are more prominent on Gettr than they are on Twitter (note that we do not have access to deleted Twitter content). If we consider the toxicity of topics, we find that topics with disproportionately high toxicity are topic 20 on race and Black Lives Matter, topic 16 on female US Democratic politicians, and topic 14 on gender issues.

No.	Topic	Representative post
1	Covid-19, Vaccines, Virus, Fauci	”More proof the jab doesn’t work as whoopiiiigasbag’s overweight triple vaxxxed ass gets hijacked by the cofraud-19!”
2	Twitter, Gettr, Facebook, Musk	”Facebook is censoring conservatives like crazy. I literally said exactly the same thing are illegitimate president said and I was banned from facebook for bullying. It’s so nuts.”
3	Ukraine, Putin, Russia, War	”Klaus Schwab and George Soros have been quite loud about this but no one is listening because they are using Ukraine as a distraction. They are also using the Ukraine invasion as a catalyst for the NWO aka great reset believe me the NWO is pure evil.”
4	Canada, Truckers, Trudeau, Convoy	”Canada is under attack from its federal and provincial governments who have been bought and sold by big pharma. ”
5	God, Lord, Jesus, Christ	”Hey Christian! What type of vessel do you desire to be for the Lord? #ChooseChristAlways”
6	Biden, Joe, Administration, President	”I have never personally seen or heard a bigger moron than Biden. He simply isn’t home. He needs to retire so the GOP can impeach hahadahoe Indian name.”
7	China, CCP, Communist, Taiwan	”This is how ccp educates young kids to be a patriot in china. It reminds me that I was brainwashed and taught since the elementary school #TakedowntheCCP #CCPisnotchines”
8	Podcast, Watch, Rogan, Episode	”Check out our latest episode of the brothers tao podcast. We discuss the #Truckersforfreedom as well as the #whoopigoldberg situation. We also take a look at the joerogan saga and what it means for censorship.”
9	Border, Illegal, Southern, Immigrants	”The absolute nerve of Biden saying we need to secure the border that he deliberately opened wide for criminal to invade our country is laughable. Actually its fkg disgusting.”
10	Election, Fraud, Audit, 2020	”Question. Would the riots at the capitol be justified if the election was stolen? You know how I know it was stolen? All the same people who told us that trump stole the 2016 election says 2020 was fair...they lie.”
11	Afghanistan, Taliban, Biden, Kabul	”Bin Laden is dead but Al Qaeda’s #2 Ayman Al-zawahri came out to celebrate the 20th anniversary of 911. It wouldn’t be so sad if the pentagon front row wasn’t filled with turncoats.”
12	Christmas, Art, Merry, Day	”My wife saw you on the warroom today and loves the color of your lipstick. She would like to know the brand and what color red.”
13	Abortions, Baby, Roe, Wade	”If a woman leaves her state where abortion is illegal and kills her baby through abortion, can she be charged with murder when she comes back home?”
14	Gender, Woman, Trans, Sex	”I have 6 male dogs, 5 are neutered, none of my dogs ever identified as a female. None went to public school either though.”
15	Freedom, Government, People, Country	”I highly suggest this article... Fight for freedom individual freedom for everybody like the constitution guarantees!”
16	Pelosi, Nancy, Woman, Hillary	”So she is saying fck the ppl and the voters. We are still passing of our agenda she’s evil and should be in prison.”
17	Supreme Court, Jackson, Scotus, Judge	”Can’t believe they nominated Jackson porno sympathizer a complete libtard.”
18	Guns, Gun Control, School, Shooting	”The government offered to buy my guns from me, but after a thorough background check of the buyer, I’m not comfortable with selling weapons to organized crime.”
19	Insurrection, Jan 6th, Antifa, Prisoners	”Keep reading updates on the abuse of the Jan 6 political prisoners and am wondering when someone in power is going to put a stop to it. Is there not one judge/law enforcement official/politician who can challenge this lawless confinement!!!??!”
20	Black, White, Racism, BLM	”Presuming or insinuating a race is oppressed gives the impression that that race is sub-par. White democrats need to stop speaking on behalf of other races. Oppression is more of a social class issue than anything, it doesn’t know race nor gender.”

TABLE I. Topics extracted from Gettr posts using BertTopic. Each topic is accompanied by a representative post, extracted automatically by BertTopic. Posts have been minorly edited for clarity and to remove unnecessary text such as URLS.

No.	Topic	Gettr %	Twitter %	Ratio
1	Covid-19, Vaccines, Virus, Fauci	16.4	10.6	1.6
2	Twitter, Gettr, Facebook, Musk	4.6	3.9	1.2
**3**	**Ukraine, Putin, Russia, War**	3.9	1.6	**2.5**
**4**	**Canada, Truckers, Trudeau, Convoy**	3.4	1.3	**2.7**
5	God, Lord, Jesus, Christ	3.2	3.7	0.9
**6**	**Biden, Joe, Administration, President**	1.8	0.7	**2.5**
7	China, CCP, Communist, Taiwan	1.6	1.0	1.6
8	Podcast, Watch, Rogan, Episode	1.4	3.0	0.5
9	Border, Illegal, Southern, Immigrants	1.4	1.0	1.4
**10**	**Election, Fraud, Audit, 2020**	1.3	0.5	**2.9**
11	Afghanistan, Taliban, Biden, Kabul	1.0	1.0	0.9
12	Christmas, Art, Merry, Day	0.9	1.4	0.6
13	Abortions, Baby, Roe, Wade	0.8	1.2	0.7
14	Gender, Woman, Trans, Sex	0.8	0.9	0.9
**15**	**Freedom, Government, People, Country**	0.8	0.3	**3.0**
16	Pelosi, Nancy, Woman, Hillary	0.7	0.5	1.4
17	Supreme Court, Jackson, Scotus, Judge	0.7	0.4	1.6
18	Guns, Gun Control, School, Shooting	0.7	0.7	1.0
19	Insurrection, Jan 6th, Antifa, Prisoners	0.7	0.4	1.9
20	Black, White, Racism, BLM	0.7	0.6	1.2
Other	N/A	14.0	22.5	0.6
Outliers	N/A	39.2	42.9	0.9

TABLE II. **Topics on Gettr and their relative prominence in the matched Twitter dataset.** Topics are listed in order of size and are characterised by a small number of keywords identified using BERTopic, see Methods. The ratio column indicates the prominence of a topic on Gettr, divided by its prominence on the matched Twitter dataset. Topics highlighted in bold are more than twice as prominent on Gettr than on Twitter. Topics not in the top 20 are grouped in the “other” category. BERTopic classifies documents as “outliers” if a topic does not correspond to a defined category.

No.	Topic	Gettr Toxicity	Twitter Toxicity
1	Covid-19, Vaccines, Virus, Fauci	0.11	0.06
2	Twitter, Gettr, Facebook, Musk	0.16	0.10
3	Ukraine, Putin, Russia, War	0.23	0.11
4	Canada, Truckers, Trudeau, Convoy	0.15	0.05
5	God, Lord, Jesus, Christ	0.10	0.06
6	Biden, Joe, Administration, President	0.34	0.18
7	China, CCP, Communist, Taiwan	0.19	0.09
8	Podcast, Watch, Rogan, Episode	0.08	0.04
9	Border, Illegal, Southern, Immigrants	0.22	0.11
10	Election, Fraud, Audit, 2020	0.15	0.05
11	Afghanistan, Taliban, Biden, Kabul	0.25	0.16
12	Christmas, Art, Merry, Day	0.06	0.05
13	Abortions, Baby, Roe, Wade	0.30	0.13
14	Gender, Woman, Trans, Sex	0.38	0.25
15	Freedom, Government, People, Country	0.20	0.10
16	Pelosi, Nancy, Woman, Hillary	0.38	0.20
17	Supreme Court, Jackson, Scotus, Judge	0.29	0.10
18	Guns, Gun Control, School, Shooting	0.23	0.12
19	Insurrection, Jan 6th, Antifa, Prisoners	0.19	0.12
20	Black, White, Racism, BLM	0.40	0.36

TABLE III. **The median toxicity of posts classified as part of each topic on Gettr and Twitter.** The Gettr toxicity is computed using all Gettr posts. The Twitter toxicity is computed using only posts from the matched cohort. On both Gettr and Twitter, the two topic with the largest toxicity are topic 20 relating to race and topic 14 relating to gender. Topics regarding Democrat politicians are also disproportionately toxic.

E. Cohort toxicity over time

In Fig. 3C of the main manuscript, we show the median toxicity of posts authored a fixed number of days after a user authored their first post on Gettr (or after their first post in our observation time window on Twitter). To rigorously assess whether post toxicity is increasing or decreasing over time, we compute a linear fit ($y = ax + b$, where a and b are slope and intercept parameters) of the normalized daily toxicity using ordinary least squares. To compute an error on the fit, we compute a bootstrapped median of each daily median toxicity 100 times using 50% of the data with replacement, resulting in 100 individual fits on the daily normalized toxicity graph. This results in median fitting parameters (95% confidence interval in square brackets) for each cohort of:

- **Non-verified:** $a = 0.0007$, $[-0.0157, 0.0139]$ units toxicity per year. $b = 0.1687$, $[0.1643, 0.1732]$.
- **Banned:** $a = -0.0091$, $[-0.0152, -0.0022]$ units toxicity per year. $b = 0.0503$, $[0.0482, 0.0530]$.
- **Matched Gettr:** $a = -0.0120$, $[-0.0137, -0.0103]$ units toxicity per year. $b = 0.0450$, $[0.0443, 0.0458]$.
- **Matched Twitter:** $a = 0.0100$, $[0.0072, 0.0127]$ units toxicity per year. $b = 0.0787$, $[0.0775, 0.0801]$.

Relative to the inter-quartile range of the post toxicity across our observation period, the median yearly change in toxicity for each cohort corresponds to:

- **Non-verified:** $0.0007/0.31 \approx 0.2\%$.
- **Banned:** $0.0091/0.13 \approx 7\%$.
- **Matched Gettr:** $0.0120/0.09 \approx 13\%$.
- **Matched Twitter:** $0.0100/0.18 \approx 6\%$.

This clearly shows that linear fits for the annual change in cohort toxicity are significantly smaller than the daily variability in post toxicity within each cohort. Consequently, we can consider the annual change in toxicity for each cohort negligible in the context of the expected fluctuations in post toxicity.

F. Quote-ratio Statistics

Statistics for the difference between the all user quote-ratio distribution shown in Fig. 5A of the main paper and each subdistribution is shown in table IV. For each statistical comparison we use a 2-sample Kolmogorov-Smirnov test to assess the difference between the test distribution and the all user distribution. The table provides the Kolmogorov-Smirnov test and corresponding p-value. A p-value less that $p = 0.01$ indicates that the test distribution is significantly different to the all user baseline. To quantify the magnitude of the difference between the distributions, we use a non-parametric analogue of the Cohen's d effect size. A verbal descriptor for the effect size is provided according to the rules of thumb in [9]. Using this terminology quantifies the difference between the all-user baseline and questionable media sources as very small (and not statistically significant). In contrast, the difference between the all-user baseline and Democrat politicians is statistically significant and described as huge according to the Cohen's d rule of thumb.

**Distribution**	**KS Statistic**	**KS p-value**	**Cohen's d**	**Cohen's d descriptor**
Matched cohort	0.22	2.8e-35	0.28	Small
Republicans	0.32	1.8e-11	0.56	Medium
Democrats	0.63	3.1e-16	2.33	Huge
Far right	0.23	0.24	0.20	Small
Right	0.21	0.002	0.43	Small
Least	0.52	1.3e-13	1.16	Large
Left	0.55	7.1e-35	1.41	Very large
Reliable	0.44	1.3e-41	1.15	Large
Questionable	0.15	0.29	0.05	Very small

TABLE IV. Statistics for the difference between the all user baseline distribution in Fig. 5 of the main paper, and each subdistribution listed in the left-most column. For each comparison we provide the KS-test statistic, the corresponding p-value, the Cohen's d effect size, and a verbal descriptor for Cohen's d, according to best practice in [9].

G. Gettr's wider impact on right-wing politics - the case of Brazil

Journalistic reports have suggested that Gettr played a key role in facilitating the Brasília insurrection on January 8, 2023, following Jair Bolsonaro's defeat in the Brazilian Presidential elections [10, 11]. Here we investigate whether there is evidence for this role in the Gettr interaction network.

First, we study the power imbalance in the Portuguese language network by measuring the Gini coefficient of the degree distribution, shown in Fig. 4A. The figure shows that the Gini coefficient peaked in the run-up to the Brasília riots, which is evidence that a handful of users were responsible for shaping the collective narrative of the Portuguese language Gettr community [12, 13].

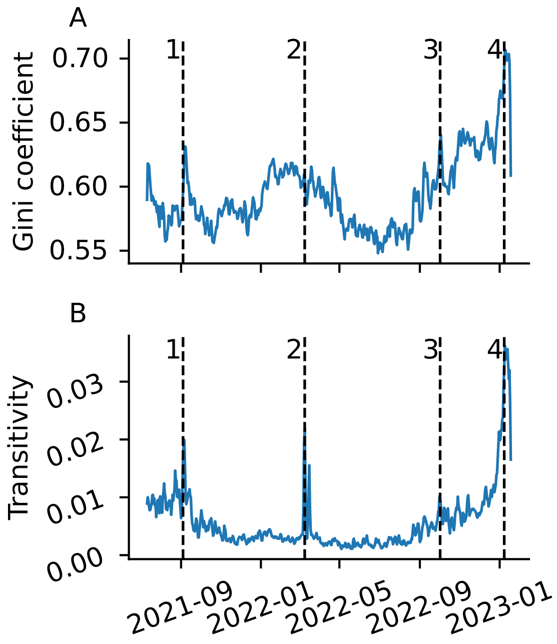

FIG. 4. **Evolution of the interaction network in the Brazilian community.** Analysis of the daily interaction network, generated by considering any interaction within a 1-day window. (A) Gini-coefficient of nodes in the giant connected component. (B) Transitivity of the giant component. Dashed lines correspond to key events related to Brazilian politics and Gettr's involvement: (1) 2021 CPAC Brazil Conference, (2) the "Ato pela terra" demonstration organized in Brasília against Bolsonaro's "Package of Destruction" laws [14], (3) the Brazilian presidential election, and (4) the Brazilian Congress attack in Brasília.

We now study grassroots engagement, measured by computing the transitivity of the Gettr interaction network, see Fig. 4B. This measure increases when a community of users densely interact with one another [15, 16]. The figure shows that network transitivity peaked during CPAC 2021, where the Bolsonaro regime and Gettr shaped their close alliance [3], and in the days leading up to the Brasília riots. Applying a Portuguese-language topic model to the network reveals that users were discussing accusations of rigged elections and claims of a corrupt media (see SI) in the lead up to the riots.

The peak in both the Gini coefficient and the transitivity shows that leading Bolsonaro allies successfully capitalised on accusations of election fraud to generate a grassroots movement on Gettr in the wake of Bolsonaro's defeat in the Brazilian elections. These results offer new quantitative insights which build on journalistic reports of Gettr's role in the riots. Critically, our results show that even when a platform appears largely inactive, a community of idle users can be mobilised within a short time period leading to real world harms. Note, however, that we cannot claim a direct link between deplatforming induced migrations to fringe social media and Gettr's potentially harmful role during the Brazilian insurrection.

H. Portuguese-language Topic Modelling

Our analysis on the Brazilian community on Gettr being focused on the impact online propaganda can have on offline upheavals, we provide the same analysis of the most salient topics mentioned by that community as we did for the English-speaking users. We use BERTopic to identify the key topics that shape the conversation within that linguistic cohort. The dominant ones are listed in table V. The topic modelling indicates that election frauds allegations are strongly discussed among the Brazilian community, which confirm the suspicions that Gettr was a key player in spreading that narrative. Notably, messages about the elections represent about 3.7% of the messages (No. 2), whereas Lula is strongly accused of stealing the elections (No. 4). Moreover, several actors who allegedly contributed in covering up the fraud, such as the mainstream media (No. 9) [17] or the Supreme Federal Court (No. 11) [18], are recurrently mentioned. Some of the key members of the Brazilian far-right circle, who are verified users on Gettr are also widely discussed within the community (No. 17). Interestingly, several of these topics are also disproportionately found within the English-speaking community (see table II). Some examples are the Covid-related conspiracy theories (No. 6), the Ukrainian war (No. 10), and the moral panic around abortion (No. 8). These findings further reinforce the overlap between the populist right in the United States and Brazil, and indicate that such narratives are platform-centric rather than bound to a specific demographic.

No.	Topic	Gettr %
1	Brazil, People, Country	14
2	Polls, Fraud, Vote, Elections	3.7
3	Lord, God, Jesus Christ, Truth	3.5
4	Thief, Bandit, Lula, Crime	3.4
5	War, Military, Patriots, Freedom	2.1
6	Health, Vaccines, Covid-19, Pfizer	1.7
7	Inflation, Salary, Economy, Budget	1.3
8	Children, Abortion, Gender, Pedophilia	1
9	Media, Press, Fake News, Journalists	1
10	Ukraine, Russia, Putin, NATO	0.9
11	Supreme Federal Court, Ministers, Constitution	0.7
12	Camp, Concentration Camp, Elderly, Children	0.6
13	Christians, Church, Pope, Satan	0.6
14	China, Communist Party, Covid-19	0.6
15	Patriots, Insiders, Terrorists, Left	0.6
16	Media, Press, Brazil, Truth	0.6
17	Jair Bolsonaro, Flavio Bolsonaro, Carla Zambelli, Carlos Jordy	0.5
Other	N/A	10.7
Outliers	N/A	54

TABLE V. **Topics which correspond to more than 0.5% of posts on Gettr in the Brazilian community.** Topics are listed in order of size and are characterised by a small number of keywords identified using BERTopic, see Methods. BERTopic classifies documents as "outliers" if a topic does not correspond to a defined category.

REFERENCES

[1] S. Ali, M. H. Saeed, E. Aldreabi, J. Blackburn, E. De Cristofaro, S. Zannettou, and G. Stringhini, in *13th ACM Web Science Conference 2021*, WebSci '21 (Association for Computing Machinery, New York, NY, USA, 2021) pp. 187–195.

[2] N. Reimann and R. Hart, "Elon musk says he's granting 'amnesty' for nearly all banned twitter accounts," `https://www.forbes.com/sites/nicholasreimann/2022/11/24/elon-musk-says-hes-granting-amnesty-for-nearly-all-banned-twitter-accounts/?sh=5d3674e72e95` Accessed 26 January, 2023 (2022).

[3] A. Maciel, "How the trump universe is backing bolsonaro's reelection bid in brazil," `https://worldcrunch.com/world-affairs/brazil-bolsonaro-trump-gettr`, Accessed 20 January, 2023 (2022).

[4] A. B. Cantor, Statistics in Medicine **20**, 2091 (2001).

[5] M. Grootendorst, ArXiv preprint 2203.05794 (2022).

[6] E. Woo, The New York Times (2022).

[7] Twitter Transparency Team, "Covid-19 misinformation transparency report," https://transparency.twitter.com/en/reports/covid19.html#2021-jul-dec (2022), accessed 7 February, 2023.
[8] K. Conger and M. Isaac, The New York Times (2021).
[9] S. S. Sawilowsky, Journal of modern applied statistical methods **8**, 26 (2009).
[10] O. Caldeira Neto, "The brazilian far-right and the path to january 8th," https://gnet-research.org/2023/01/23/the-brazilian-far-right-and-the-path-to-january-8th/, Accessed 24 January, 2023 (2023).
[11] C. Ecarma, "The right-wing media's coverage of brazil's insurrection is a rerun of january 6," https://www.vanityfair.com/news/2023/01/right-wing-media-brazils-insurrection Accessed 24 January, 2023 (2023).
[12] L. Zhu and K. Lerman, "Attention inequality in social media," (2016).
[13] B. Guinaudeau, F. Vottax, and K. Munger, Unpublished paper. Available at: https://osf. io/f7ehq/download (2020).
[14] J. Rocha, "Brazilians vs bolsonaro's 'package of destruction'," https://lab.org.uk/brazilians-vs-bolsonaros-package-of-destruction/ Accessed 25 July, 2023 (2022).
[15] M. Falkenberg, Communications Physics **4**, 200 (2021).
[16] K. Orman, V. Labatut, and H. Cherifi, Complex Networks , 99 (2013).
[17] M. Camarotto, "Despite efforts to fight falsehoods, brazil's tight election is threatened by dangerous lies," https://reutersinstitute.politics.ox.ac.uk/news/despite-efforts-fight-falsehoods-brazils-tight-election-threatened-dangerous-lies Accessed 17 March, 2023 (2022).
[18] R. Brito and C. Pulice, "Bolsonaro challenges brazil election he lost to lula," https://www.reuters.com/world/americas/brazils-bolsonaro-files-complaint-challenge-election-results-2022-11-22/ Accessed 17 March, 2023 (2022).
